# Retrograde posterior column acetabular screw: robotically assisted and CT-guided minimally invasive procedure

**DOI:** 10.1007/s00113-026-01704-z

**Published:** 2026-04-07

**Authors:** Dominik M. Haida, Friedemann Meiswinkel, Georgi Prosenikov, Stefan Huber-Wagner

**Affiliations:** 1 Klinikum rechts der Isar, Department of Trauma Surgery, Technical University of Munich, Ismaninger Straße 22, 81675 Munich, Germany; 2 Department of Trauma Surgery, Diak Klinikum Landkreis Schwäbisch Hall, Diakoniestraße 10, 74523 Schwäbisch Hall, Germany

**Keywords:** Robotics, 3D mixed reality glasses, Pelvis, Acetabulum, Robotic arm, Robotik, 3‑D-Mixed-Reality-Brillen, Becken, Acetabulum, Roboterarm

## Abstract

**Objective of the surgery:**

The aim of this operation is to stabilise a T-type acetabular fracture against secondary dislocation and to restore the patientʼs preoperative functionality.

**Indications:**

An 88-year-old patient with severe pain and immobility due to a T-type acetabular fracture (Letournel & Judet: associated fracture; AO: 62B2).

**Contraindications:**

General surgical contraindications.

**Surgical technique:**

This surgery was performed in a 3D-Navigation Hybrid Operating Room, the Robotic Suite (Brainlab, Munich, Germany). The setup includes a navigation unit (Curve Navigation System) a movable robotic 3D cone beam computed tomography unit (Loop-X), a robotic arm (Cirq Arm System), 3D mixed reality glasses (for planning), and a wall monitor (Buzz).

A step-by-step video is available online.

**Follow-up:**

Immediate full weight-bearing, active and passive joint mobilisation through physiotherapy on the first postoperative day, pain medication as required.

**Evidence:**

Navigated surgeries of the pelvis (ring and acetabulum) have become standard practice, while robotic assistance is increasingly used, demonstrating very good outcomes and promising accuracy rates.

**Video online:**

In the online version of this article (10.1007/s00113-026-01704-z), you will find an informative video on the surgical technique presented (© courtesy of the authors, all rights reserved).

## Abstract (de)

**Operationsziel:**

Das Ziel dieser Operation ist es, eine acetabuläre T‑Fraktur gegen eine sekundäre Dislokation zu sichern und den präoperativen Funktionszustand des Patienten wiederherzustellen.

**Indikation:**

88 Jahre alter Patient mit sehr starken Schmerzen und Immobilität sowie einer acetabulären T‑Fraktur (Letournel & Judet: kombinierte Fraktur; AO: 62B2).

**Kontraindikationen:**

Allgemeine Kontraindikationen für eine Operation.

**Op.-Technik:**

Diese Operation wurde in einem 3‑D-Navigations-Hybrid-OP durchgeführt, der „Robotic Suite“ (Fa. Brainlab, München, Deutschland). Diese besteht aus der Navigationseinheit „Curve Navigation System“, einem fahrbaren robotischen 3‑D-Cone-Beam-CT (CBCT) „Loop-X“, einem robotischen Arm „Cirq Arm System“, 3‑D-Mixed-Reality-Brillen (zur Planung) und dem Wandmonitor „BUZZ“.

**Weiterbehandlung:**

Sofortige Vollbelastung, aktive und passive Gelenkmobilisation durch Physiotherapie am ersten postoperativen Tag, Schmerzmedikation bei Bedarf.

**Evidenz:**

Navigierte Techniken sind bereits etabliert am Becken (Beckenring und Acetabulum), robotische Assistenzsysteme werden zunehmend eingesetzt und zeigen dabei vielversprechende Operationsergebnisse und eine hohe Genauigkeit.

## Background

Navigation and robotics have become an important part of orthopaedic trauma surgery in recent years [11]. This development has also extended to pelvic surgery. While navigation is widely used in pelvic and acetabular surgery [1, 13], robotically assisted surgery is less common [8, 18, 20]. Within orthopaedics, spinal surgery currently leads the way in the use of robotics due to its achievements and promising future potential [17]. The advancement of robotic surgery has enabled high accuracy rates in pedicle screw placement at specific levels in the spine as well as facilitating minimally invasive approaches [7, 9].

This potential also exists in acetabular surgery, where the screw trajectory can be narrow and a variety of approaches can be chosen [19]. When performing open reduction and internal fixation (ORIF) procedures at the acetabulum, it is still important to consider that visibility and accessibility often need to be weighed against the invasiveness of the procedure when choosing the approach [19].

It is now up to surgeons to transfer proven conventional and navigated techniques to the sphere of robotically assisted pelvic and acetabular surgery. The aim is to achieve accuracy rates comparable to those established in spinal surgery and perform minimally invasive surgery, ultimately leading to good patient outcomes.

With this article, we aim to contribute to this promising development. To the best of our knowledge, there is no description in the literature of robotically assisted placement of a retrograde posterior column screw at the acetabulum.

We intend to highlight the feasibility and benefits of a robotically assisted and minimally invasive placement of a retrograde posterior column screw at the acetabulum. Older patients in particular can benefit from this procedure due to its minimally invasive nature combined with safe and highly accurate robotically assisted screw placement.

## Definitions and classifications

The most common classifications used for acetabular fractures are those of Letournel and Judet [10, 12] as well as the AO Foundation/Orthopaedic Trauma Association (AO/OTA) classification for acetabular fractures [14].

Letournel and Judet differed in their classification between five types of elementary fractures and five types of associated fractures [10, 12]. In elementary fractures, only a single wall or column of the acetabulum is involved, whereas associated fractures affect at least two structures (walls or columns).

The AO/OTA classification applies its standard A, B, and C differentiation for fracture types to acetabular fractures [14, 16]. A‑type fractures are partially articular and involve a single column or wall [16]. In addition to an A‑type fracture, B‑type fractures include a transverse fracture component. C‑type fractures are completely intra-articular and involve both columns.

A wide range of approaches are available for surgery of the acetabulum. A general distinction is made between anterior, posterior, extended, and combined approaches [19]. There are currently three preferred approaches, which include the Kocher ilioinguinal approach (anterior), the Kocher–Langenbeck approach (posterior approach), and extended approaches (executed as an extended iliofemoral approach or modified Maryland approach; [19]).

The choice of acetabular stabilisation technique depends on the selected approach and on the available resources, such as navigation or robotic systems. A percutaneous and therefore minimally invasive retrograde posterior column screw can be placed via an approach through the tuber ischiadicum [2].

## Case

A physically fit and healthy 88-year-old male patient fell onto his buttocks at home while getting undressed. Due to persisting severe pain and immobility, he presented to the emergency room of our hospital the following day.

Imaging (X-ray and computed tomography) revealed a left-sided T‑type acetabular fracture (AO: 62B2) along with an anterior pelvic ring fracture of the superior left pubic ramus.

## Surgical indication

When determining the indication for surgery in the case of an acetabular fracture, the following points must be considered: joint stability, joint congruity, additional injuries to the acetabulum, and the general condition of the patient [15].

In our case, considering the patient’s age (88 years), the high level of pre-injury functionality, and the accompanying pain (visual analogue scale of 8) combined with a T-type fracture (associated fracture according to Letournel and Judet; [10, 12]), the decision was made together with the patient in favor of surgical treatment.

After assessing all risk factors, the most suitable option was to treat the posterior column with robotically assisted, minimally invasive, retrograde posterior column screw fixation. The goal was to stabilise the fracture against secondary dislocation, reduce pain, and restore mobility. The anterior pelvic ring fracture was treated conservatively.

## Surgical technique (see also video online, Fig. 1)

Our hospital is equipped with a 3D Navigation Hybrid Operating Room (OR) consisting of a navigation unit (Curve Navigation System), a movable robotic 3D cone beam computed tomography (CBCT) system (Loop-X), a robotic arm (Cirq Arm System), and a wall monitor (Buzz; all components: Brainlab, Munich, Germany). Interventions at the spine, pelvis (pelvic ring and acetabulum), and extremities are regularly performed in this Hybrid OR [4–8].

**Fig. 1:**
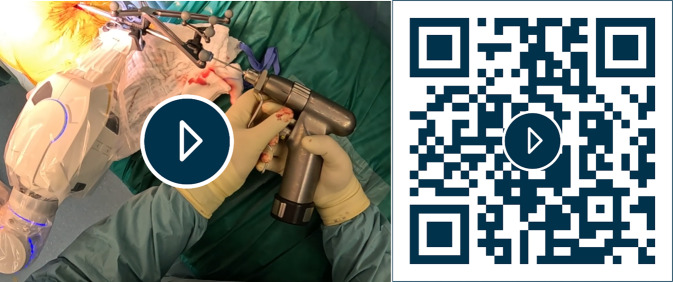
A supplementary video is available for this article. Scan the QR code or click on the link provided to view the video online: https://www.springermedizin.de/link/10.1007/s00113-026-01704-z/#supplementary-content

**Fig. 2:**
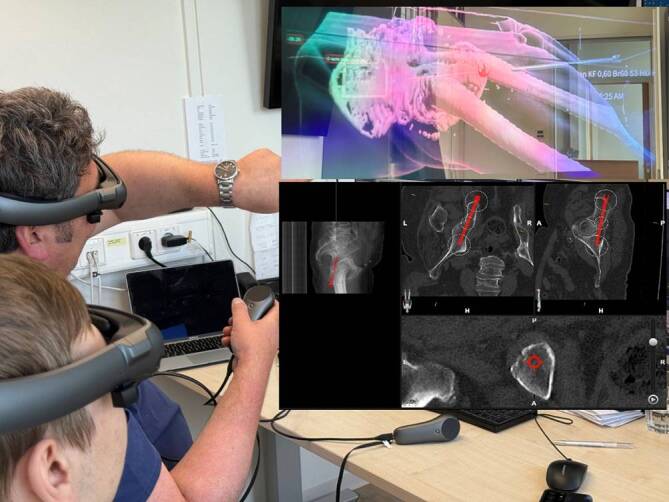
Proof of feasibility using 3D mixed reality glasses. *Blue trajectory*: entry point; *red trajectory*: planned screw trajectory

Prior to the operation, the feasibility of this procedure was proven with the help of 3D mixed reality glasses (Elements Mixed Reality Viewer, Brainlab; Fig. 2). This process also included planning the patient positioning due to the integration of the robotic arm (00:34 min).

In the OR, the patient is placed (00:42 min) in the prone position on a carbon fibre operating table, with positioning pads elevating the pelvis and the legs spread apart. The legs point toward the CBCT. After careful disinfection (which is very important due to the proximity of the anus) and complete multi-layer draping, the intervention area is prepared for surgery.

The operation begins with the placement of the reference array close to the intervention area in order to achieve high accuracy (00:53 min). Two pins are inserted into the posterior iliac crest and the reference star is attached. It is important to maintain visual contact between the camera and the reference array for the entire duration of the operation.

The first CBCT scan is prepared. The intervention area is covered with sterile drapes. The CBCT is then moved into position (01:14 min) and a field image is obtained to ensure adequate visualisation of the area of interest (01:20 min). The table and CBCT positions are saved for subsequent scans. If the field image is satisfactory, the collision check (01:33 min) and the actual 3D CBCT scan (01:37 min) can then be performed. (Note: The patient is preoxygenated with 100% oxygen prior to the scan. The scan is performed during apnea, and the OR team exits the room for the duration of the scan.)

**Fig. 3:**
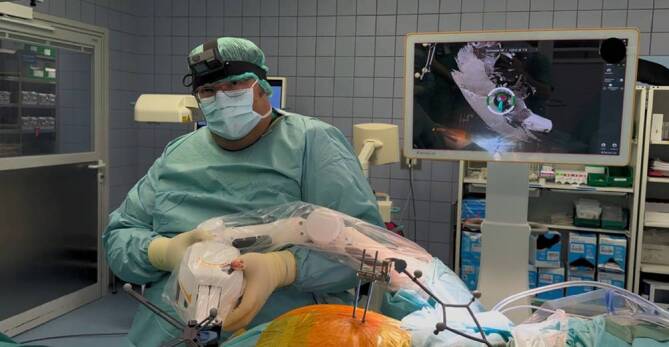
Preparation of the robotically assisted drilling

After the scan is performed, its accuracy is verified visually using the pointer (01:44 min). If the scan is accurate, screw planning is then carried out manually with the help of the pointer and on the navigation screen (01:48 min). The initial screw is planned with dimensions of 7.5 mm × 120 mm (diameter × length). After screw planning, the skin incision is marked using the pointer (02:37 min). Once the skin incision is made, the instruments (e.g., the drill guide) are registered in the system (02:53 min). The surgeon then guides the robotic arm towards the planned trajectory while observing the navigation screen and pressing both black buttons located at the head of the robotic arm (03:09 min; Fig. 3). When the trajectory on the screen lights up green, the surgeon can release both buttons and the robotic arm approaches the final position autonomously (03:12 min).

The drilling guide is inserted and guided down to the bone using dissecting scissors to hold the soft tissue aside (03:25 min). The surgeon may now perform minor fine adjustments while receiving visual feedback from the navigation screen. If the trajectory of the drilling guide aligns with the planned screw, the guide is lightly tapped onto the bone to secure it against slipping (03:40 min). This is followed by the drilling and insertion of a K-wire (03:50 min).

After inserting the K‑wire, the table and the CBCT are returned to the positions saved from the first CBCT scan in order to perform the second 3D CBCT scan (04:39 min). After the second scan is completed, the position of the inserted K‑wire is checked on the navigation screen in multiple planes (04:45 min). It is now possible to determine the final screw length by precise measurement on the screen. After the measurement, the decision is made to insert a cannulated and fully threaded 7.5 mm × 115 mm screw (Königsee Implantate, Allendorf, Germany). For the screw placement, the K‑wire is overdrilled (05:16 min) and the screw is inserted (05:20 min).

The final position of the screw is confirmed with a 2D X‑ray from the CBCT in two planes, showing proper placement (05:26 min). The K‑wire is then removed and the wound is closed (05:34 min).

The postoperative X‑ray and CT scan show the screw in a very satisfactory position (05:38 min). Mobilisation was possible as early as on the second postoperative day with the aid of a walker (05:59 min).

## Postoperative treatment

**Fig. 4:**
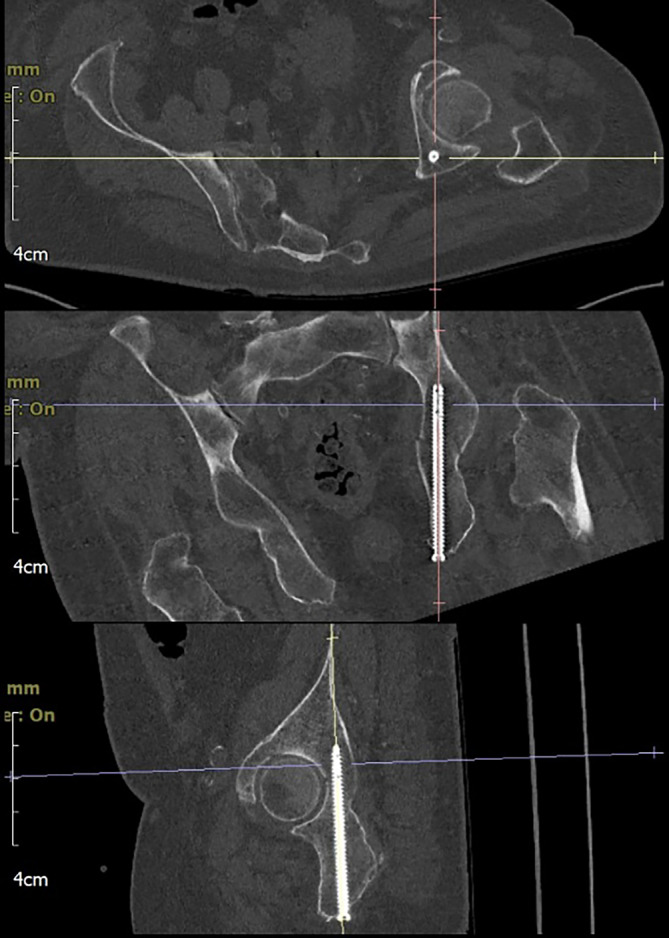
Postoperative multiplanar CT of the retrograde posterior column acetabular screw

Full weight-bearing was permitted immediately after the operation. Active and passive joint mobilisation was initiated through physiotherapy. Pain medication according to the WHO stage scheme was given as required. Mobilisation was possible as early as on the second postoperative day with the aid of a walker. The implant position was checked prior to discharge (X-ray and CT; Fig. 4). The patient was discharged on the sixth postoperative day to a geriatric rehabilitation facility. Further radiographic follow-up was recommended at 6 and 12 weeks postoperatively. Implant removal is not intended.

## Pitfalls, risks, and complications

There are various aspects to consider when working in a Hybrid OR.

It is essential that all team members receive sufficient and adequate training in the proper handling of the technical equipment. In this context, structured workflows are crucial.

To ensure accurate navigation data throughout the procedure, imaging correctness should always be verified manually using the pointer on known anatomical structures and confirming the visual feedback on the navigation screen.

When drilling the holes for the K‑wire, it is important to do so under visual guidance on the navigation screen, to ensure drilling is performed exactly along the planned screw trajectory. In areas with pronounced soft tissue, precise drilling can be facilitated by tapping the drilling sleeve firmly onto the bone to prevent slipping.

## Evidence of the technique

As this is the first description of a robotically assisted retrograde posterior column acetabular screw, there is no literature available.

However, navigation and modern imaging solutions have long been used in pelvic surgery (mainly pelvic ring) and are well established [1, 11, 13, 15]. By contrast, robotically assisted surgery of the pelvis and acetabulum is relatively new but shows great potential, enabling minimally invasive approaches combined with high accuracy rates [3, 7, 18, 20].

## Practical conclusion

- Modern imaging solutions can reduce radiation exposure to the surgical team.
- The accuracy of intraoperative cone beam computed tomography (CBCT) scans should always be verified manually using a pointer.
- Checking the wire position with a CBCT scan enables high accuracy in screw placement.
- Modern imaging solutions in combination with advanced navigation software allow for precise screw selection.
- Robotically assisted surgery of the pelvis (pelvic ring and acetabulum) can be performed minimally invasively with high accuracy rates.
- With this procedure, early mobilisation can be achieved in older patients.
- Robotically assisted, minimally invasive placement of a retrograde posterior column screw at the acetabulum is highly feasible.

## Supplementary Information

ESM1: Supplementary material 1

## Declarations

This article does not contain any studies involving humans or animals. All patients who can be identified from images or other information within the manuscript have given their written consent.
